# Double burden of malnutrition as a risk factor for overweight and obesity

**DOI:** 10.11606/s1518-8787.2022056004205

**Published:** 2022-11-03

**Authors:** Antonio Bernabé-Ortiz, Carmen Quinteros-Reyes, Rodrigo M. Carrillo-Larco

**Affiliations:** I Universidad Peruana Cayetano Heredia Center of Excellence in Chronic Diseases Lima Peru Universidad Peruana Cayetano Heredia. Center of Excellence in Chronic Diseases. Lima, Peru; II Universidad Científica del Sur Lima Peru Universidad Científica del Sur. Lima, Peru; III Imperial College London School of Public Health Department of Epidemiology and Biostatistics London UK Imperial College London. School of Public Health. Department of Epidemiology and Biostatistics. London, UK

**Keywords:** Child, Malnutrition, Risk Factors, Overweight, Obesity, Maternal and Child Health

## Abstract

**OBJECTIVE:**

To assess the longitudinal effect of double burden of malnutrition (DBM) on the risk of developing child and adolescent overweight or obesity.

**METHODS:**

Analysis of the Peruvian Young Lives Study, younger cohort: baseline (2002) and 4 follow-ups (2006–2007, 2009–2010, 2013–2014, and 2016–2017). Outcomes were the incidence of overweight and obesity as defined by the World Health Organization standards. The exposure comprised a variable with 4 categories: non-stunted child with a non-overweight mother (reference group), non-stunted child with an overweight mother, stunted child with a non-overweight mother, and stunted child with an overweight mother (i.e., DBM). Poisson regression models were built to assess the association of interest, and relative risks (RR) and 95%CI were reported.

**RESULTS:**

Data from 2,034 children; 50.0% were girls and the mean age was 12.0 (3.6) months at baseline. Non-stunted children with an overweight mother had greater risk (RR = 1.64; 95%CI: 1.35–1.99) of developing overweight, compared with the risk for stunted children with a non-overweight mother (RR = 1.38; 95%CI: 1.10–1.72), and for those with DBM (RR = 1.28; 95%CI: 1.02–1.61). When compared with the reference group, obesity risk was greater among non-stunted children with an overweight mother (RR = 2.33; 95%CI: 1.68–3.22), greater among stunted children with a non-overweight mother (RR = 2.59; 95%CI: 1.75–3.84), and greater among those with DBM (RR = 2.14; 95%CI: 1.39–3.28).

**CONCLUSIONS:**

DBM is a risk factor for childhood overweight and obesity in Peru. Dual-duty policies tackling both undernutrition in children and overweight in mothers are needed to reduce DBM and its future effects in Peru.

## INTRODUCTION

The trends of a rising body mass index (BMI) among children and adolescents have reached a plateau in different high-income countries but started to hasten in low- and middle-income countries (LMIC)^1^. Nevertheless, the BMI changes over time are highly variable across countries, highlighting distinct nutritional quality and different exposure to health risks during life^2^.

The double burden of malnutrition (DBM), a condition defined as the coexistence of overnutrition and undernutrition, usually in mother and child, has emerged as a health concern in LMIC^3^. Such coexistence may be present at the individual level (stunted children with obesity), household level (stunted children with overweight mother), and at population level (high prevalence of anemia and obesity in the same country)^4,5^. According to the World Health Organization (WHO) and other reports^6,7^, the DBM at the household level is the more common form of DBM in middle-income countries undergoing rapid nutrition transition. Worldwide, the prevalence of DBM within household ranged from 0% to 26.8% by country^8^. The exposure to early undernutrition (i.e., stunting or famine) is widely documented as having negative effects on child health^9^, with increases in overweight and obesity rates^10^ and subsequent emergence of non-communicable conditions^11,12^. Thus, one hypothesis considers that intrauterine growth retardation, low birth weight, and premature birth have a causal relationship to the origins of non-communicable conditions such as overweight and obesity (Barker hypothesis)^13^. On the other hand, the thrifty gene hypothesis proposes that some genes determine the increase of fat storage, which represent a survival advantage in times of famine, but may result in obesity in a non-famine environment^14^. Studies, however, have shown, separately, the effect of both conditions (undernutrition and obesity) on child health, resulting in limited evidence on the effect of the DBM on overweight and obesity during childhood.

Peru has seen a significant reduction in the prevalence of children with stunting, especially in the urban settings when compared with rural (average annual percent change of -4.5% *versus* -2.6%, respectively)^15,16^. Overweight and obesity rates, however, have increased among mothers since 1992^16^. Nevertheless, the composition of the DBM has almost not changed during the last 30 years: 90% of children with DBM in Peru are stunted^17^. Although relevant, much of this information is descriptive and comes from analyses of repeated Peruvian Demographic Health Surveys, instead of longitudinal data on the same subjects.

Currently, Peru is going through the nutrition transition, offering a unique scenario to study the potential effect of DBM on child health. Consequently, we aim to evaluate the longitudinal effect of the DBM and the risk of developing future overweight or obesity using an ongoing cohort of children transitioning to adolescence. We hypothesize that this association would vary due to child undernutrition and mother overweight status, being greater in the group with DBM.

## METHODS

### Study Design

Analysis of the Peruvian data of the Young Lives Study. The Young Lives Study is an ongoing prospective cohort carried out in four LMIC (Ethiopia, India, Peru, and Vietnam)^18^. This cohort started in 2002 and completed five evaluations: the baseline and four rounds of follow-up.

### Study Participants

In Peru, the Young Lives Study comprises two different cohorts: the younger cohort that enrolled 2,052 children from the ages of 6 to 18 months at baseline, and the older cohort that included 1,000 children from the ages of 7 to 8 years. The baseline (2002), and the first (2006–2007), second (2009–2010), third (2013–2014), and fourth (2016–2017) follow-ups of the younger cohort were used for analysis. This decision was taken to include children with early undernutrition (i.e., in the first 1,000 days of life). Figure shows a flowchart of the study and sample size in each of the study assessments.

**Figure:**
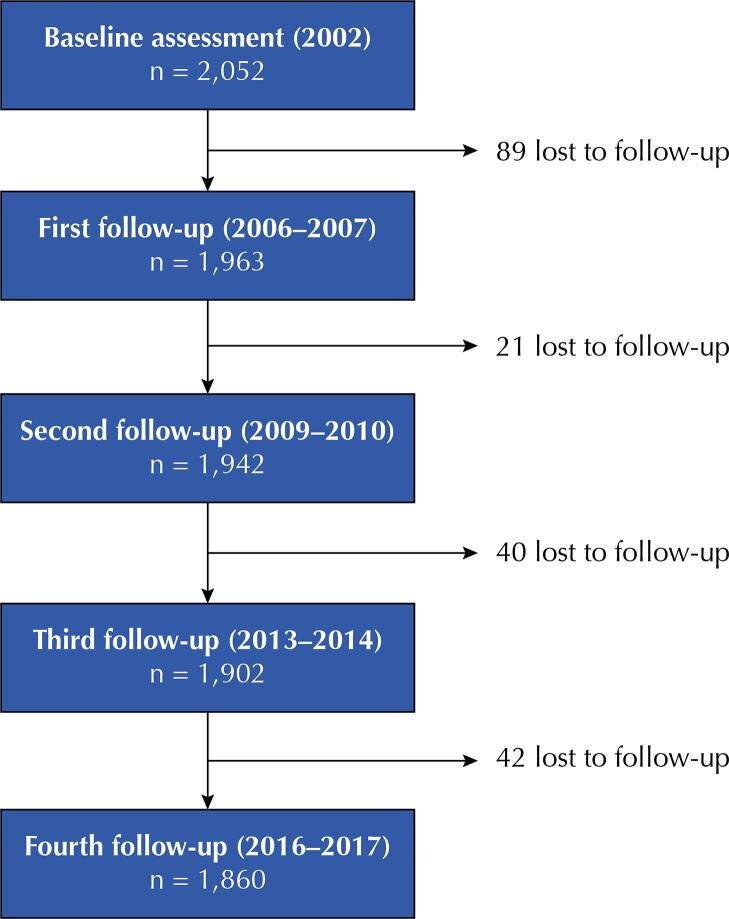
Flowchart of the younger cohort from the Young Lives Study in Peru.

### Sampling Strategy

The Young Lives Study used a sentinel site sampling approach as described in a previous study^19^. Briefly, the sampling strategy was based upon a multistage, cluster stratified, random sampling technique. The sample frame was conducted at the district level with a random selection of 20 sentinel sites from a total of 1,518 districts available in Peru. Since an oversampling of poor areas was required, the top 5% richest districts were excluded from the sampling approach.

Furthermore, the maps of census tracts, with blocks or sets of houses, were used to randomly select one census tract in each district using a table of random numbers. Finally, all households in any given block or set of houses were visited to identify one household with at least one child accomplishing the inclusion criteria for study purposes. Different blocks or sets of houses were approached until the total eligible households were completed. For data collection, three teams – comprising of fieldworkers, a data-entry clerk, and supervisors – were responsible for six or seven sentinel sites.

### Study Variables

Outcome: The outcomes of interest were the incidence of overweight and obesity, both defined using the BMI according to the WHO standards^20^. Overweight was defined as ≥ 2 standard deviations (SD) above the median of the BMI-for-age, if the child was < 5 years of age, or ≥ 1 SD above the median of the BMI-for-age if the child was ≥ 5 years of age; whereas obesity was defined as ≥ 3 SD above the median of the BMI-for-age if the child was < 5 years of age, or ≥ 2 SD above the median of the BMI-for-age if the was child ≥ 5 years of age. Child weight and height data were available for all the assessments.

Exposure: The exposure of interest was DBM defined, for our purposes, as a stunted child with an overweight mother. A child was considered stunted whether they had a height-to-age z-score < 2 SD below the average z-score, according to the WHO standards^21^. If a mother had a BMI ≥ 25 kg/m^2^, she was considered overweight^22^. A variable was created by combining anthropometrical markers from both children and mother and then split into 4 categories: non-stunted child with a non-overweight mother (reference group in regression models), non-stunted child with an overweight mother, stunted child with a non-overweight mother, and stunted child with an overweight mother. This categorization was done to explore differential risk between nutrition categories as pointed out by our initial hypothesis.

Covariates: Other variables were also included in the analysis as potential confounders. Variables at the child level include sex (male *versus* female); age (in months) in each assessment; whether the child was born by cesarean delivery, reported by the mother (no *versus* yes); and if it was premature at birth, reported by the mother (no *versus* yes). Other variables were at the mother level, such as maternal age (in years) and maternal education level (< 7, 7–11, and ≥ 12 years). Variables at the household level were socioeconomic status, based on a wealth index, created as a simple average of housing quality (rooms in the household, main material of walls, roofs, floor, and the number of people living in the dwelling), access to services (electricity, drinking water, sanitation facility, and cooking fuel), and consumer durables (automobile, blender, bicycle, iron, etc.)^23^, which was finally split into tertiles (low, middle and high) for analysis, natural region (coast, highlands, and jungle), and area type (urban *versus* rural).

### Statistical Analysis

Stata 16 for Windows (StataCorp, College Station, TX, US) was used to analyze data. Initially, the study population was described at baseline according to DBM categories. Means and standard deviation (SD) for numerical variables, and proportion and frequencies for categorical variables were used. Comparisons were performed with analysis of variances or Chi-square test, depending on whether it was a numerical or categorical variable.

The changes in the proportion of stunted children, overweight and obese mothers, and DBM categories were tabulated using a bivariate approach. Incidence and 95% confidence intervals (95%CI) for overweight and obesity were also estimated. To assess the association of interest – i.e., whether DBM is a risk factor for future overweight and obesity – crude and adjusted models were built using Poisson regression considering the repetitive assessment (panel data) of variables and the multilevel design of the study using random intercepts. The first level was defined by the child’s characteristics and its different evaluations over time, whereas in the second level, the sentinel site was included to account for potential clustering as participants in one cluster are more similar compared to other different clusters. All the models were controlled for potential confounders, and relative risks (RR) and 95%CI were reported. Finally, differences between DBM categories (i.e., non-stunted children with non-overweight mother *versus* stunted children with non-overweight mother *versus* stunted children with overweight mother) was conducted using Wald test of simple and composite linear hypothesis about the parameters of the multivariable model, and p-values were reported when appropriate.

### Ethics

The Young Lives Study protocol and informed consent was approved by the Ethics Committee Social Science Division, University of Oxford, UK, in 2002. Informed consent was obtained from all participants. In Peru, the approvals were granted by the Research Ethics Committee of the Nutrition Research Institute in Lima, Peru. Data is freely available under request and information is de-identified to guarantee confidentiality and anonymity of the original participants. All methods were carried out in accordance with relevant guidelines and regulations.

## RESULTS

### Characteristics of the Study Population at Baseline

A total of 2,052 children were originally recruited in the younger cohort in Peru, of which 18 were excluded due to incomplete data in the variables of interest. Therefore, we included 2,034 children, 1,017 (50.0%) females, with a mean age of 12.0 (3.6) months. A total of 623 (30.6%) non-stunted children had an overweight mother, whereas 392 (19.3%) stunted children had a mother without overweight. Finally, 184 (9.1%; 95%CI: 7.8–10.4) children were stunted and had an overweight mother, compatible with the usual definition of DBM (Table 1).

**Table 1:** Characteristics of the study population at baseline.

	Non-stunted child/ non-overweight mother	Non-stunted child/ overweight mother	Stunted child/ non-overweight mother	Stunted child/ overweight mother	p
			
	(n = 835)	(n = 623)	(n = 392)	(n = 184)	
Child sex					< 0.001
Female	444 (53.2%)	327 (52.5%)	155 (39.5%)	91 (49.5%)	
Child age (months)					< 0.001
Mean (SD)	11.6 (3.5)	11.6 (3.5)	13.2 (3.4)	13.1 (3.5)	
C-section delivery					< 0.001
Yes	84 (10.1%)	122 (19.6%)	24 (6.1%)	24 (13.0%)	
Premature at birth					0.08
Yes	229 (27.7%)	179 (29.1%)	84 (22.1%)	52 (29.1%)	
Child BMI					< 0.001
Mean (SD)	17.7 (1.9)	18.2 (1.9)	17.5 (2.0)	18.2 (3.5)	
Child BMI Z-score					< 0.001
Mean (SD)	0.7 (1.2)	1.0 (1.2)	0.7 (1.3)	1.0 (1.9)	
Maternal age (years)					< 0.001
Mean (SD)	25.1 (6.0)	28.2 (6.5)	26.6 (7.1)	30.3 (7.6)	
Maternal education level (years)					< 0.001
≤ 6	324 (40.6%)	182 (30.4%)	259 (71.5%)	103 (58.9%)	
7–11	317 (39.8%)	262 (43.8%)	78 (21.6%)	52 (29.7%)	
≥ 12	156 (19.6%)	154 (25.8%)	25 (6.9%)	20 (11.4%)	
Socioeconomic level					< 0.001
Low	295 (35.4%)	106 (17.0%)	209 (53.3%)	68 (37.0%)	
Middle	270 (32.3%)	207 (33.2%)	130 (33.2%)	73 (39.7%)	
High	270 (32.3%)	310 (49.8%)	53 (13.5%)	43 (23.3%)	
Region					< 0.001
Coast	286 (34.3%)	335 (53.8%)	43 (11.0%)	41 (22.3%)	
Highlands	395 (47.3%)	226 (36.3%)	287 (73.2%)	119 (64.7%)	
Jungle	154 (18.4%)	62 (9.9%)	62 (15.8%)	24 (13.0%)	
Area type					< 0.001
Rural	280 (33.6%)	100 (16.1%)	228 (58.3%)	76 (41.3%)	

SD: standard deviation; BMI: body mass index.

### Changes of Double Burden of Malnutrition Over Time

Overall, the proportion of stunted children is decreasing over time, from 28.3% at baseline to 16.1% at the last follow-up (Table 2). The proportion of mother with overweight and obesity increased in the same period, from 30.8% to 43.7% for overweight, and from 8.9% to 36.0% for obesity. Regarding DBM categories, the proportion of children living with an overweight mother increased from 30.6% to 67.6% in the case of non-stunted children, and from 9.0% to 12.1% in the case of stunted children (Table 2).

**Table 2:** Changes of child stunting, mother overweight and double burden of malnutrition over time.

	Baseline	Follow-ups			

		First	Second	Third	Fourth
				
	(n = 2,040)	(n = 1,954)	(n = 1,938)	(n = 1,878)	(n = 1,843)
Child stunted					
Yes	587 (28.3%)	651 (33.3%)	402 (20.7%)	358 (19.1%)	296 (16.1%)
Maternal BMI					
Normal	1,228 (60.3%)	830 (44.9%)	643 (35.0%)	391 (23.5%)	314 (20.3%)
Overweight	627 (30.8%)	737 (39.8%)	791 (43.1%)	754 (45.3%)	677 (43.7%)
Obese	181 (8.9%)	284 (15.3%)	403 (21.9%)	518 (31.2%)	559 (36.0%)
Double burden of malnutrition					
Non-stunted child/ non-overweight mother	835 (41.1%)	509 (27.6%)	471 (25.7%)	291 (17.6%)	252 (16.4%)
Non-stunted child/overweight mother	623 (30.6%)	735 (39.8%)	984 (53.7%)	1,060 (64.0%)	1,038 (67.6%)
Stunted child/ non-overweight mother	392 (19.3%)	319 (17.3%)	171 (9.3%)	97 (5.9%)	60 (3.9%)
Stunted child / overweight mother	184 (9.0%)	282 (15.3%)	208 (11.3%)	208 (12.5%)	186 (12.1%)

BMI: body mass index.

### DBM as a Risk Factor for Future Child Overweight and Obesity

During the 14.0 (SD = 0.5) years of follow-up, the incidence of childhood overweight was 7.9 (95%CI: 7.6–8.2) per 100 person-year. Such incidence was 5.4 (95%CI: 4.9–6.0) among non-stunted children with non-overweight mother, 10.2 (95%CI: 9.8–10.7) among non-stunted children with an overweight mother, 4.6 (95%CI: 3.8–5.4) among stunted children with a non-overweight mother, and 5.1 (95%CI: 4.5–5.9) among those with DBM.

The incidence of childhood obesity was 2.2 (95%CI: 2.0–2.4) per 100 person-year of follow-up, and incidence estimates were 1.1 (95%CI: 0.8–1.5) for non-stunted children with non-overweight mother, 3.2 (95%CI: 2.9–3.5) for non-stunted children with an overweight mother, 1.1 (95%CI: 0.8–1.5) among stunted children with non-overweight mother, and 2.1 (95%CI: 1.6–2.7) among those with DBM.

The multivariable model showed evidence that non-stunted children with an overweight mother had 64% (95%CI: 35–99) greater risk of developing overweight when compared with non-stunted children with non-overweight mother, and this risk was higher (p-value = 0.03) if compared with the 38% (95%CI: 10–72) risk for stunted children with a non-overweight mother and the 28% (95%CI: 2–61) for those with DBM. Nevertheless, there was no difference between the latter two risk estimates (p = 0.31).

Similarly, when compared with the reference group, the evidence showed that the risk for future obesity was 133% (95%CI: 68–222) greater among those non-stunted children with an overweight mother, 159% (95%CI: 75–284) greater among stunted children with a non-overweight mother, and 114% (95%CI: 39–228) greater among those with DBM (Table 3). The three risk estimates were not different between them (p > 0.05).

**Table 3:** DBM as a risk factor for overweight and obesity: crude and adjusted models.

	Crude model	Adjusted model^a^
	
	RR (95%CI)	RR (95%CI)
For child overweight		
Child non-stunted/non-overweight mother	1 (Reference)	1 (Reference)
Child non-stunted/overweight mother	1.67 (1.44–1.93)	1.64 (1.35–1.99)
Child stunted/ non-overweight mother	1.13 (0.88–1.46)	1.38 (1.10–1.72)
Child stunted/overweight mother	1.13 (0.92–1.39)	1.28 (1.02–1.61)
For child obesity		
Child non-stunted/non-overweight mother	1 (Reference)	1 (Reference)
Child non-stunted/overweight mother	1.96 (1.52–2.53)	2.33 (1.68–3.22)
Child stunted/ non-overweight mother	1.59 (1.07–2.35)	2.59 (1.75–3.84)
Child stunted/overweight mother	1.50 (1.01–2.22)	2.14 (1.39–3.28)

^a^ Adjusted for child sex, child age, C-section delivery, child premature at birth, maternal age, maternal education level, socioeconomic level, region, and are type.

DBM: double burden of malnutrition; RR; relative risks; 95%CI: 95% confidence intervals.

## DISCUSSION

### Main Findings

Although the results did not support our initial hypothesis, our findings shows that DBM at childhood has a strong association with the risk of developing overweight and obesity. Thus, in the case of overweight, the risk was lower among stunted children (having or not having an overweight mother) compared with non-stunted children with an overweight mother, whereas there was no difference in the increased risk for obesity.

### Comparison with Previous Studies

Our study expands current literature by using a wide definition of DBM and assessing the potential differential effects of children stunting and mother overweight. Thus, within our model we could determine the joint effect of stunting in children when having an overweight mother, but also the effect of only child stunting. Literature regarding this topic is scarce, especially in Latin America, where most of the manuscripts are focused on cross-sectional analyses reporting changes in the DBM over time^17,24^. A cross-sectional study in Peru evaluated data from children under-5 years of age and their mothers, using information from the Demographic Health Surveys from 1996 to 2016, and reported that during this period there was an important increase in the proportion of children with and without undernutrition that have an overweight or obese mother^17^. We found similar results, suggesting the importance of understanding why overweight and obesity in mothers has increased while undernutrition in their children has decreased over time.

Early undernutrition exposure, including stunting and famine, has been associated with future poor health outcomes^9^. Thus, stunting has been associated with irreversible long-term effects on health, including cognitive impairment, reduced physical growth potential and birth weight of offspring, and increased risk of diet-related non communicable diseases^5^. A systematic review reported that famine exposure during early life increased the mean BMI as well as the risk of overweight and obesity^27^. In the same line, overweight, type 2 diabetes, hyperglycemia, metabolic syndrome, and schizophrenia were more common among adults born during the famine in China compared with those born after the famine^28^. It is expected that early nutrition impairment may have an effect on future organ size, structure, and function, affecting diverse hormonal axes regulating growth and appetite, and influencing on both the risk and the metabolic effects of subsequent overweight and obesity^9,29^. Nevertheless, scarce literature has used DBM to assess its effect on the risk of overweight and obesity.

### Public Health Relevance

Our results show that different factors should be considered when assessing the risk of child overweight and obesity. In the case of non-stunted children with an overweight mother, the environment seems to be relevant as children are in an obesogenic niche, which may affect diet and physical activity patterns^30^. In the case of stunted children, regardless of the mother’s BMI status, the effect of malnutrition on child overweight and obesity is clear but lower than that of the non-stunted child with an overweight mother group. As undernutrition may affect different hormonal axes^9^, a delay in child development might explain these findings^31^. Moreover, despite of the obesogenic niche, stunted children with an overweight mother still have a potentially long way to go from undernutrition to obesity.

Additionally, our findings enhance the importance to address DBM in Peru through the development of dual-duty policies to avoid unintended effects of one-sided programs^9,32^. Our findings suggest that, despite the risk DBM represents for future overweight and obesity among children, non-stunted children with an overweight mother (a condition that is increasing over time) are at an even greater risk of developing overweight and obesity. In Peru, actions to prevent malnutrition are one-sided policies to reduce undernutrition. Although it is not possible to attribute the great reduction of stunting in Peru to any program alone, the CRECER strategy, which includes food assistance programs (FAP) that provide access and increase food consumption, has been considered as one of the catalysts for that improvement^33,34^. However, the effect of FAP in the same Young Lives cohort showed that mothers have a 93% risk of developing obesity compared with those who were not exposed to FAP; and that, even though children exposed to FAP showed a low risk of obesity, these programs tend to provide excess of carbohydrates and few servings of fruits, which could result in overnutrition of mothers and children^35^. This shows how the unintended consequences of one-sided policies like FAP could affect the quality of the diet; thus, accelerating the rapid weight gain and increasing the risk of developing obesity^9 32^. Therefore, dual-duty policies that simultaneously address all forms of malnutrition^32^, for both children and mothers, must be developed to combat the DBM and the future risk of overweight and obesity in children.

### Strengths and Limitations

Our analysis used data from a well-known long-term cohort study with information from the first year of life until adolescence. Our models considered the repetitive nature of several variables, including DBM, child and maternal age, and socioeconomic position. Our outcome was evaluated as categorical variable to ensure appropriate interpretation of findings. Notwithstanding, this study also has limitations that should be highlighted. First, selection bias may be an issue since 5% of the richest districts were excluded according to the sampling strategy. Moreover, changes in BMI and overweight and obesity rates are initially seen in wealthiest individuals among countries undergoing nutrition transition, which may hinder the inference of our results. Second, we only used the definition of DBM at the household level in detriment of any other definition. However, this kind of DBM has been recognized as more common in countries such as Peru. Third, some variables, potential confounders, were not included in the analyses since they were not available (e.g., diet patterns, including fruit and vegetable intake or junk food consumption, physical activity, among others). Fourth, the attrition rate, although small (< 10%), may have affected our results. Finally, other noncommunicable conditions, such as hypertension or hyperglycemia were not available, limiting the analysis to only overweight and obesity. Nevertheless, our findings are relevant to understand the potential long-term effect of the DBM on health markers.

## CONCLUSIONS

Our findings show strong association between childhood DBM and the risk of adolescent overweight and obesity. Our results suggest the need of interventions tackling both children undernutrition and overweight in mothers, known as dual-duty policies.
